# Resveratrol and Montelukast Alleviate Paraquat-Induced Hepatic Injury in Mice: Modulation of Oxidative Stress, Inflammation, and Apoptosis

**DOI:** 10.1155/2017/9396425

**Published:** 2017-10-22

**Authors:** Noha A. El-Boghdady, Nourtan F. Abdeltawab, Mohammed M. Nooh

**Affiliations:** ^1^Department of Biochemistry, Faculty of Pharmacy, Cairo University, Cairo, Egypt; ^2^Department of Microbiology and Immunology, Faculty of Pharmacy, Cairo University, Cairo, Egypt

## Abstract

Paraquat (PQ) is one of the most used herbicide worldwide. Its cytotoxicity is attributed to reactive radical generation. Resveratrol (Res) and montelukast (MK) have anti-inflammatory and antioxidant properties. The protective effects of Res, MK, or their combination against PQ-induced acute liver injury have not been investigated before. Therefore, we explored the protective potential of Res and/or MK against PQ hepatic toxicity in a mouse model. Mice were randomly assigned to five groups: group I served as the normal control and group II received a single dose of PQ (50 mg/kg, i.p.). Groups III, IV, and V received PQ plus oral Res (5 mg/kg/day), MK (10 mg/kg/day), and Res/MK combination, respectively. Res and/or MK reduced PQ-induced liver injury, evidenced by normalization of serum total protein, ALT, and AST. Res and/or MK significantly reversed PQ-induced oxidative stress markers glutathione and malondialdehyde. Res and/or MK significantly reduced PQ-induced inflammation reflected in TNF-*α* levels. Furthermore, Res and/or MK reversed PQ-induced apoptosis assessed by differential expression of *p53*, *Bax*, and *Bcl-2*. Histopathologic examination supported the biochemical findings. Although Res and MK displayed antioxidative, anti-inflammatory, and antiapoptotic activities, their combination was not always synergistic.

## 1. Introduction

Poisoning by chemicals used in agriculture is a critical public health problem worldwide particularly in developing countries. Paraquat (PQ) is one of the top herbicides used in agriculture that is highly toxic for humans and animals. Although the lung is the primary organ affected, PQ toxicity affects other organs such as the liver [1]. The liver is the main site for xenobiotic metabolism and has a high potential for generating reactive oxygen species (ROS). Thus, it is at high risk for toxic damage [2]. In fact, the liver has been regarded as a key target for PQ poisoning [3].

The toxicity of PQ stems from its induction of ROS and the subsequent induction of oxidative stress [4]. After entering cells, PQ is reduced to an unstable radical that is then reoxidized to form a cation and generates a superoxide anion, hydroxyl free radical, and peroxynitrite [5]. Although several studies examined PQ-induced toxicity, few addressed the apoptotic and the transcriptional regulatory mechanisms as potential contributory factors or the importance of modulating these machineries in treating PQ poisonings. Furthermore, there is no specific antidote or effective treatment for PQ poisoning that has been identified so far; thus, new treatment strategies should be sought. Using an agent with multiple potentials, for example, antioxidant, anti-inflammatory, and antiapoptotic properties, would have a favorable value in the treatment of PQ-induced liver injury.

Resveratrol (Res, trans-3,4′,5-trihydroxystilbene) is a small polyphenol compound found in more than 70 plant species, including berries, nuts, and grapes [6]. Over the years, this molecule has received considerable attention for its anti-inflammatory properties [7], antioxidant properties [8], and general health-improving ability in mammals [9]. Pharmacokinetic and pharmacodynamic studies have demonstrated that the liver and kidney are main target organs for Res [10]. Res exerts a hepatoprotective activity in ethanol-, thioacetamide-, and ischemia reperfusion-induced liver damage [11–13]. Also, Res exhibits an immune modulatory effect by suppressing overproduction of inflammatory cytokines like TNF-*α*, IL-1*β*, and IL-6 [14]. Systemic administration of Res has been shown to inhibit the initiation and growth of tumors in a wide variety of cancer types [15]. Although the beneficial properties of Res have been well known, the mechanisms by which Res protects the liver against the cytotoxic effects of PQ are not well defined.

Montelukast (MK) is a leukotriene receptor antagonist with anti-inflammatory and antioxidant properties [16, 17]. Cysteinyl leukotrienes (CysLTs) are formed by inflammatory cells, such as mast cells, eosinophils, and basophils. They are potent proinflammatory mediators that increase microvascular permeability and are effective chemotactic agents [18, 19]. CysLT receptors are present in the airways, liver, and other organs [20]. CysLT1 antagonists, such as MK, have been reported to ameliorate experimental colitis [21], burn- and sepsis-induced multiorgan damage [22, 23], and renal ischemia reperfusion-induced oxidative damage [24]. MK acts by inhibiting neutrophil infiltration, balancing oxidant-antioxidant status, and controlling inflammatory mediator generation [25].

To the best of our knowledge, there is no report regarding the protective effects of MK or its combination with Res against PQ-induced acute liver injury. Therefore, the current study aimed to examine the protective potential of Res, MK, and both on PQ-induced hepatic damage by exploring the hepatic antioxidant, anti-inflammatory, histopathologic, and antiapoptotic events in a mouse model.

## 2. Materials and Methods

### 2.1. Chemicals and Drugs

PQ dichloride and Res were purchased from Sigma (St. Louis, MO, USA). MK was from Merck Sharp & Dohme Ltd. (Hoddesdon, Hertfordshire, UK). All other chemicals and reagents used were of the highest analytical grade and were obtained from Sigma.

### 2.2. Animals

Male Swiss albino mice weighing 25–35 g were obtained from the animal house of the Faculty of Medicine, Cairo University, Egypt. They were allowed one week for acclimatization before starting the experiment. The animals were kept under controlled conditions (25 ± 2°C temperature with 12 h light/dark cycle) and were allowed standard chow and water ad libitum during the entire experiment. All efforts were made to minimize animal suffering, if any, during the experiment.

The current study protocol was approved by the Ethics Committee for Animal Experimentation at the Faculty of Pharmacy, Cairo University (reference number: PT 1287). All animal procedures complied with the “Guide for the Care and Use of Laboratory Animals” (US National Institutes of Health publication no. 85–23, revised 1996).

### 2.3. Experimental Design

Animals were randomly divided into five groups. Group I (normal control (NC) group; *n* = 10) received a single intraperitoneal injection of normal saline and oral distilled water daily. Group II (PQ group; *n* = 14) received a single intraperitoneal injection of PQ dissolved in normal saline (50 mg/kg) with no treatment for the subsequent two days. PQ dose was shown to induce liver damage in mice [26] and was chosen after preliminary studies. Group III (Res group; *n* = 12) received Res (5 mg/kg/day; in distilled water by oral gavage) [27] for five consecutive days, three days before and two days after PQ injection. Group IV (MK group; *n* = 12) received MK (10 mg/kg/day; in distilled water by oral gavage) [28] using the same regimen as that for group III. Group V (Res+MK group; *n* = 12) received a combination of both Res (5 mg/kg/day, p.o.) and MK (10 mg/kg/day, p.o.) using the same regimen as that for group III. After PQ injection, animals were monitored for any adverse outcomes, and those showing signs of health deterioration during the study were euthanized.

### 2.4. Blood Collection and Sample Analysis

Three hours after the last treatment, mice were anaesthetized for blood collection from the retro-orbital sinus and serum was separated, aliquoted, and stored at −80°C till determination of liver function tests. Serum activity of alanine transaminase (ALT) and aspartate transaminase (AST) were estimated using kits provided by Quimica Clinica Aplicada (Spain) according to the manufacturer's instructions. Serum protein level was measured using a kit supplied by Bio-Diagnostic (Egypt) according to the manufacturer's instructions.

### 2.5. Tissue Preparation and Sample Analysis

Immediately after blood collection, the mice were euthanized and livers were rapidly removed, washed with ice-cold saline, blot-dried, and weighed. Sections from the liver were taken for histopathological examination, tumor necrosis factor-*α* (TNF-*α*) measurement, and RNA isolation. The remaining tissue was used to prepare 20% homogenate in ice-cold double distilled water. The obtained liver homogenate was aliquoted and immediately frozen at −80°C for biochemical analysis.

### 2.6. Determination of Liver MDA and GSH Levels

An aliquot of the liver homogenate was mixed with an equal volume of 2.3% ice-cold KCl and another aliquot with 7.5% sulfosalicylic acid for the determination of MDA and GSH levels, respectively. The contents were mixed well and centrifuged at 600 ×g at 4°C for 15 min. The MDA level, an indicator for products of lipid peroxidation, was assayed by monitoring thiobarbituric acid reactive substance formation by the spectrophotometric method described by Mihara and Uchiyama [29]. Lipid peroxidation was expressed as nmol MDA/g tissue. GSH was determined by the spectrophotometric method based on the use of 5,5′-dithiobis-2-nitrobenzoic acid as described by Beutler et al. [30], and the results were expressed as mg GSH/g tissue.

### 2.7. Determination of Hepatic TNF-*α* Levels

About 50 mg of the liver was weighed and homogenized in a suitable volume of the lysis buffer. The lysate was centrifuged at 10,000 × g for 15 min at 4°C. The supernatant was used for the quantitation of TNF-*α* level using an ELISA assay (Quantikine mouse TNF-*α* kit; R&D Systems Inc., Minneapolis, Minnesota, USA) according to the manufacturer's instructions. The concentration of TNF-*α* was expressed as pg/mg protein [31].

### 2.8. Determination of Protein Content in Liver Homogenate

The protein content of the TNF-*α* fraction, resulting from ultracentrifugation of liver homogenate was determined by the method of Lowry et al. [32] using bovine serum albumin as standard.

### 2.9. Gene Expression Analysis (qRT-PCR)

Total RNA was extracted from liver tissues using RNeasy Mini kit (Qiagen, CA, USA), and the quality and quantity of the obtained RNA was assessed spectrophotometrically. Equal amounts of RNA were reverse transcribed into cDNA using QuantiTect Reverse Transcription kit (Qiagen, CA, USA) according to the manufacturer's instructions. To assess the expression of target genes, quantitative real-time PCR was performed using Rotor-Gene SYBR Green PCR kit (Qiagen, CA, USA) with Rotor-Gene Q system (Qiagen, CA, USA). *β*-Actin (Actb) was used as a housekeeping reference gene. The sense and antisense primers were as follows: *β-actin*, 5′-CTAAGGCCAACCGTGAAAAG-3′ (sense) and 5′-ACCAGAGGCATACAGGGACA-3′ (antisense); B-cell lymphoma 2 (*Bcl-2*), 5′-AGTACCTGAACCGGCATCTG-3′ (sense) and 5′-GGGGCCATATAGTTCCACAAA-3′ (antisense); Bcl-2-associated X protein (*Bax*), 5′-GTGAGCGGCTGCTTGTCT-3′ (sense) and 5′-GGTCCCGAAGTAGGAGAGGA-3′ (antisense); and cellular tumor antigen p53 (*p53*), 5′-ATTAAAGGATGCCCGTGCT-3′ (sense) and 5′-GGCCCTTCTTGGTCTTCG-3′ (antisense). PCR reactions included 5 min enzyme activation at 95°C, followed by 40 cycles with 95°C for 5 sec (denaturing) and 60°C for 10 sec (annealing/extension). The changes in target genes expression were calculated using comparative CT (ΔΔCT, threshold cycle) method and presented as fold change using the 2^−ΔΔCT^ formula [33].

### 2.10. Histological Examination

Liver specimens were fixed in 10% formaldehyde and subsequently embedded in paraffin and sliced into slices of 4 *μ*m thickness followed by staining with hematoxylin and eosin (H&E) and examined under light microscope [34].

### 2.11. Statistical Analysis

The results were expressed as the mean ± SEM and statistical comparisons were carried out using one-way analysis of variance (ANOVA), followed by Tukey's multiple comparison test. Statistical analysis was performed using GraphPad Prism software (version 5). *p* < 0.05 was taken as the minimal level of significance.

## 3. Results

### 3.1. Effect of Res, MK, and Their Combination on PQ-Induced Hepatic Injury

PQ resulted in substantial elevation in serum ALT (24%) and AST (20%) activity along with a decrease in serum protein level (31%) as compared to the control mice (Table 1). These observations reflected generalized tissue damage. Administration of Res, MK, and their combination reversed the deleterious effects of PQ (Table 1).

### 3.2. Effect of Res, MK, and Their Combination on Oxidative Stress Markers

**Figure 1:**
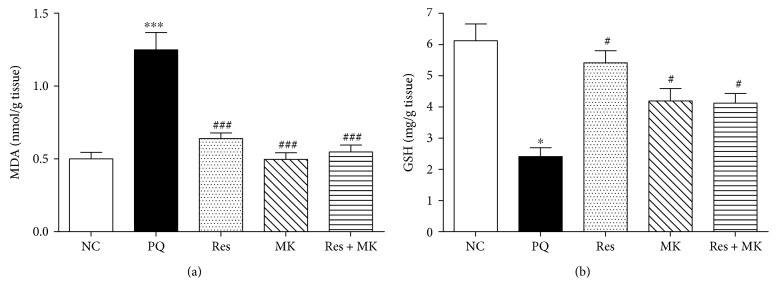
Effect of Res, MK, and Res+MK on hepatic oxidative stress markers. (a) MDA and (b) GSH levels. Data represent the mean ± SEM (*n* = 10–14). ∗ and ∗∗∗ mean significantly different from the control group at *p* < 0.05 and *p* < 0.001, respectively. # and ### mean significantly different from the PQ-treated group at *p* < 0.05 and *p* < 0.001, respectively.

Malondialdehyde (MDA), a stable metabolite of the free radical-mediated lipid peroxidation cascade, is widely used as a marker of oxidative stress. Hepatic MDA level was considerably increased in PQ-treated group by 2.5-fold as compared to the NC group. However, administration of Res, MK, and their combination markedly reduced MDA level (49%, 60%, and 56%, resp., as compared with the PQ group) (Figure 1(a)).

Oxidative stress is an imbalance between oxidant generation and antioxidant system including reduced glutathione (GSH), a radical scavenger. Hepatic GSH content was substantially decreased in PQ group (63%) as compared with the normal mice. But, Res, MK, and their combination caused a marked increase in the hepatic GSH content (2.25-, 1.7-, and 1.7-fold, resp., as compared with the PQ group) (Figure 1(b)).

### 3.3. Effect of Res, MK, and Their Combination on Hepatic TNF-*α* Level

**Figure 2:**
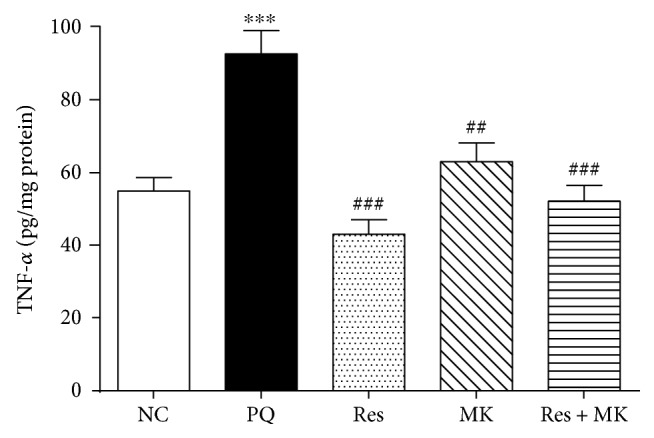
Effect of Res, MK, and Res+MK on hepatic TNF-*α* level. Data represent the mean ± SEM (*n* = 10 − 14). ∗∗∗ significantly different from the control group at *p* < 0.001. ## and ### mean significantly different from the PQ-treated group at *p* < 0.01 and *p* < 0.001, respectively.

PQ injection considerably increased hepatic TNF-*α* level by 68% as compared to the normal group. However, this effect was abolished by treatment with Res, MK, and their combination (53%, 32%, and 44%, resp., as compared with the PQ group) (Figure 2).

### 3.4. Effect of Res, MK, and Their Combination on Expression of Apoptosis Markers

**Figure 3:**
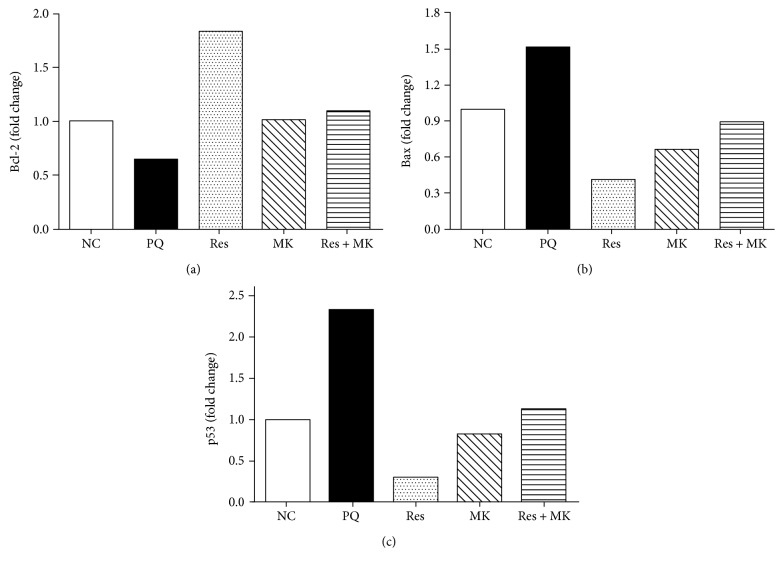
Effect of Res, MK or Res+MK on mRNA expression of *Bcl-2*, *Bax*, and *p53* genes. Data represent the mean of technical duplicates of the pooled total RNA from *n* = 5. (a) B cell lymphoma-2 (*Bcl-2*), (b) Bcl-2 associated x protein (*Bax*), and (c) cellular tumor antigen *p53* mRNA expression was detected by quantitative RT-PCR.

During the pathogenesis of acute PQ poisoning, exposure of hepatocytes to intracellular stressors such as ROS may trigger apoptosis [35]. Thus, we investigated whether Res, MK, or their combination can suppress apoptosis. This was addressed via assessing expression of *Bcl-2*, *Bax*, and *p53*. As observed in Figure 3, exposure to PQ triggered apoptosis as indicated by increased expression of the proapoptotic *p53* (2.3-fold) and *Bax* (1.5-fold) together with downregulation of *Bcl-2*, an antiapoptotic gene. Interestingly, administration of Res, MK, and their combination counteracted these changes in favor of cell survival suggesting that these agents can protect the hepatocytes from apoptosis.

### 3.5. Effect of Res, MK, and Their Combination on Hepatic Histopathological Changes

**Figure 4:**
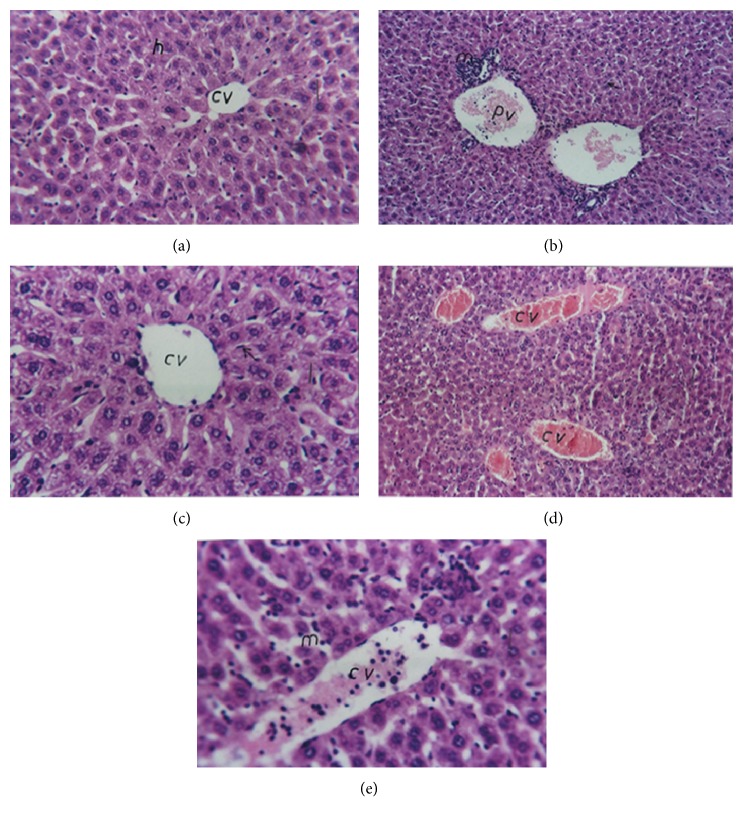
Photomicrographs of H&E stained liver sections from (a) normal control mice (64x), normal architecture of central vein (CV), and surrounding hepatocytes (h). (b) PQ group (40x): sever dilatation in portal vein (PV) with inflammatory cell infiltration (m) in the portal area surrounding the bile duct. (c) Res group (80x): dilatation of CV with diffuse Kupffer cells proliferation in between the hepatocytes (arrow). (d) MK group (40x): dilatation and congestion in the CV. (e) Res+MK group (80x): dilatation of CV with inflammatory cell infiltration in between the hepatocytes (m).

Normal histological structure of the central vein and surrounding hepatocytes was observed in normal control mice (Figure 4(a)). By contrast, liver sections from mice receiving PQ showed severe dilatation in the portal vein associated with inflammatory cell infiltration in the portal area surrounding the bile ducts (Figure 4(b)). Administration of Res, MK, and their combination ameliorated the alterations in liver morphology as evidenced by mild congestion in the central vein (Figures 4(c), 4(d), and 4(e), resp.). Res succeeded in mitigating Kupffer cell proliferation in between the hepatocytes; however, inflammatory cell infiltrations in between the hepatocytes were still observed in MK and Res+MK groups. A semiquantitative assessment of the severity of reaction in the liver according to histopathological alterations was shown in Table 2.

## 4. Discussion

The metabolic and histological features in acute PQ intoxication are similar in humans and mice [36]. In the current study, PQ induced liver injury in mice as evidenced by histopathological changes, significant increase in serum ALT and AST, and decrease in serum protein levels. The hepatocellular damage triggered by PQ may be through the observed elevation of oxidative stress, inflammation, and apoptosis. Therefore, it was attractive to investigate whether MK and/or Res would be effective against PQ-induced liver damage in mice.

The cytotoxic actions of PQ have been shown to involve oxidative stress with subsequent inflammatory responses and apoptosis [37]. Monitoring ROS-induced modifications of cellular constituents and changes in antioxidant systems is commonly employed for evaluating oxidative stress. The primary line of defense against ROS is GSH that is usually exhausted by tissue-damaging agents [1, 16]. GSH is vital for cell metabolism, differentiation, and proliferation [38]. Approaches directed to preserving GSH content may be implicated in the conservation of cellular integrity. On the other hand, MDA is one of the most commonly used indicators of lipid peroxidation: the process that has been implicated in several deleterious effects such as increased membrane rigidity, osmotic fragility, and reduced mitochondrial survival [39]. We and others demonstrated lipid peroxidation as a biomarker of PQ hepatic toxicity [37, 40, 41]. It was demonstrated that Res can modulate cellular redox in several models of liver injury [11–13] and in subacute PQ toxicity in mice [41]. Similarly, MK has been shown to diminish oxidative stress markers and enhance antioxidants in rat liver [16, 17, 42]. In the present study and in agreement with the previous studies, Res and MK abolished MDA and elevated GSH levels; however, their effects were not synergistic when combined.

Proinflammatory cytokines play a pivotal role in organ damage and oxidative status [16, 42]. Inflammatory cytokines, particularly TNF-*α*, chemokines, and growth factors have been shown to be involved in the pathogenesis of PQ toxicity [43]. In accordance with previous studies, our findings demonstrated that PQ elevated hepatic TNF-*α* level. Moreover, the ability of Res to reduce PQ-induced elevation of hepatic TNF-*α* level may partly account for its protective effects. Similarly, Res was reported to decrease TNF-*α* level that may be through blocking the activation of nuclear factor kappaB (NF-*κ*B) and activator protein-1 (AP-1) [44]. On the other hand, MK is a well-established antiasthmatic drug that can reduce cytokine levels and prevent inflammation [16, 45]. Therefore, it was expected to suppress PQ-induced inflammation as reflected by our findings.

Apart from the inflammatory response and oxidative stress, apoptosis may be another culprit in the hepatotoxic events of PQ poisoning [46]. In the current study, three apoptosis-related genes, Bax, Bcl-2, and p53, were investigated. The proapoptotic gene Bax is one of the members of the Bcl-2 gene family. It is a dominant inhibitor of Bcl-2 that is another member of the Bcl-2 family. Bcl-2 prevents apoptosis by cleaning up ROS and preventing the release of Ca^2+^ from the endoplasmic reticulum [47]. The 53-kDa transcription factor p53 is constitutively expressed at low levels in most cells and tissues [48]. It is presumably the most extensively studied apoptosis marker, since its enhanced expression induces apoptosis [48]. We and others demonstrated that PQ markedly upregulated hepatic p53 expression [49, 50]. It was revealed that PQ-induced ROS and TNF-*α* were important activators of p53 expression through their capacity to induce DNA strand breaks [48] and the induction of the NF-*κ*B [51], respectively. Consistent with our results, Res was shown to enhance and suppress the expression of Bcl-2 and of Bax, respectively, in several organs [52–54]. Previous studies explored the possible involvement of leukotriene receptor blockade by MK in apoptosis [55–57]. In accordance with these observations, the current findings supported the antiapoptotic effect of MK. Thus, it is possible that agents with antioxidant and anti-inflammatory properties could also provide protective and antiapoptotic effects against tissue damage.

Using two agents with antioxidant, anti-inflammatory, and antiapoptotic properties in combination, such as Res and MK, was expected to show an additive effect. However, in the present study, the combination of both compounds did not produce superior improvements as compared to the effect of each drug separately. Such lack of synergy may be attributed to the selected doses used in the study or the short duration of treatment. Another possibility is that the magnitude of antioxidant and anti-inflammatory effects reached a plateau with each molecule by itself. Thus, this combination does not favor a greater change. Also, a potential limitation of our study is that we did not establish whether concurrent occurrence of both drugs in the gut could modify their individual absorption or metabolism. Furthermore, other Res combinations reported in the literature have also shown a nonsynergistic effect [58, 59].

Findings of the present study demonstrated that Res, MK or their combination can protect liver against the PQ-induced injury as they could suppress ROS generation, inflammation, and apoptosis triggered by PQ. This was evidenced by the significant amelioration in the altered histological observations parallel to the diminished serum ALT and AST activities. However, the combination of Res and MK did not demonstrate any additional benefit than each individual treatment. Further studies are necessary to evaluate the therapeutic effects of Res and MK in clinical uses.

## Figures and Tables

**Table 1:** Effect of Res, MK, and their combination (Res+MK) on serum ALT, AST, and total protein.

Parameters	Groups				
	NC	PQ	Res	MK	Res+MK
AST (U/L)	64.4 ± 0.95	77.7 ± 3.84^∗^	64.8 ± 2.67^#^	57.2 ± 4.21^##^	51.1 ± 3.24 ^∗^^,###^
ALT (U/L)	33.3 ± 2.1	41.2 ± 1^∗^	25.3 ± 1.5 ^∗^^,###^	30.5 ± 2.4^##^	25.3 ± 0.95 ^∗^^,###^
Protein (g/dL)	6.25 ± 0.29	4.33 ± 0.25^∗∗∗^	5.55 ± 0.28^#^	6.05 ± 0.2^##^	5.63 ± 0.36^#^

Data are expressed as mean ± SEM (*n* = 10–14); ∗and ∗∗∗ versus control group at *p* < 0.05 and *p* < 0.001, respectively. #, ##, and ### versus PQ-treated group at *p* < 0.05, *p* < 0.01, and *p* < 0.001, respectively.

**Table 2:** Semiquantitative assessment of the severity of reaction in the liver according to histopathological alterations.

Histopathological alterations	Groups				
	NC	PQ	Res	MK	Res+MK
Congestion in PV	−	+++	−	−	−
Congestion in CV	−	−	+	+	+
Diffuse Kupffer cell proliferation	−	−	+	−	−
Inflammatory cell infiltration in portal area	−	+++	−	−	−
Diffuse inflammatory cell infiltration in between hepatocytes	−	−	−	++	+++

–: nil; +: mild; ++: moderate; +++: severe.

## Additional Points

*Availability of Data and Materials.* The data supporting the conclusions of this article are included within the article.
